# Book Reviews

**Published:** 1821-03

**Authors:** 


### CRITICAL ANALYSES

OF

### RECENT PUBLICATIONS, IN THE DIFFERENT BRANCHES OF MEDICINE AND SURGERY.

"I would have men know, that, though I reprehend the easie passing over of the causes of things "by ascribing them to secret and hidden vertues and properties; (for this hath arrested and laid "asleepe all true enquiry and indications;) yet I doe not understand but that, in the practical "part of knowledge, much will be left to experience and probation, whereunto indication cannot "so fully reach: and this not only in *specie*, but in *individuo*. Yet it was well said, *Vere scire* "*esse per causas scire*."—BACON.

*Practical Observations on the Colchicum Autumnale, as a general Remedy of great Power in the Treatment of Inflammatory Diseases, both acute and chronic; and therefore as a Substitute for Bleeding, in Disorders which are connected with increased Action of the Heart and Arteries.* By CHARLES THOMAS HADEN, Esq. Surgeon to the Chelsea and Brompton Dispensary; late Surgeon to the Derbyshire General Hospital; Member of the Medico-Chirurgical and other Societies. 8vo. pp. 84. Burgess and Hill, London. 1820.

THE powerful agency of colchicum in gout and rheumatism, has for some years been so generally admitted by medical practitioners in this part of the world, that, however common it may be to write what has been better written before, few persons, we believe, would think of gravely announcing its utility in these complaints, as a matter of novelty. Mr. HADEN, accordingly, lays claim to originality on different grounds: first, as having introduced a new form of exhibition; and, secondly, of having discovered in colchicum the power of producing certain effects on the sanguiferous system. "In both these particulars, therefore, the writer claims for this pamphlet the praise of containing something that is new and useful."

Occasional mention is made of colchicum by the ancient writers both of Greece and Rome, by whom it was ranked among the poisons; but, we believe, the earliest particular account of its properties was given by PAULUS ÆGINETA, who regarded it as a remedy of great efficacy in gout, the symptoms of which, he asserts, were generally removed by it within two days. He goes on to say, "I also knew a man who prescribed hermodactylus, not according to the usual forms and in substance, but he boiled the plant with anise and parsley, and gave the decoction to be drank." DEMETRIUS PAPAGOMENUS, who wrote in the thirteenth century, likewise particularly mentions it; a translation of one of whose formulæ we subjoin:

℞. Aloes . . . . ʒj.

Hermodactyli . ʒss.

Cinnamomi . . ʒjss.

Scammoniæ . . gr. x.

Ex iis fiant pilulæ, dentur pro viribus materiæ copia.

Mr. Kerr (to whose "Medical Sketches" we refer our readers for some curious information on the early history of this drug,) goes on to remark: "In this prescription we have no account of the exact preparation of each of the ingredients, nor of the medium to be used in forming them into pills. Most probably, the dry root of the hermodactylus was powdered, and some simple addition made to the aloes, that the whole might be made into a ductile mass."

In the reign of Queen Elizabeth, a translation of Wertzung's "Praxis Medicinæ Universalis" was published, in which a particular description is given of this plant, and very favourable mention made of its virtues. More recently we find it enjoying its reputation, and entering into the "Pulvis Arthriticus" of Sir Theodore Mayerne, in which it was combined, among other *et cætera*, with the powder of unburied skulls, (*cranii humani insepulti*;) an ingredient which, it would appear, was not much relished by his Majesty King James the First; for it is added in a note, "N.B. In casu D. N. Regis qui *ανθρωποφαγους* odit, cranium humanum poterit in ossium bubulorum rasuram permutari." What alteration the efficacy of the nostrum suffered from this change, we are not informed.

From the foregoing remarks and quotations, it appears that colchicum has been used in medicine from a very early period, and *generally in the solid form;* so that, if there be any novelty in our present practice, it consists in our having discontinued its exhibition in that state: and Mr. Haden has, therefore, the merit of recalling our attention to the manner in which this drug was given in former times.

So much for the form of exhibition. A more important question, however, is to ascertain whether Mr. Haden be or be not right in regarding colchicum "as a remedy of great efficacy in controling the action of the heart and arteries, *and therefore* as a substitute for the lancet in the treatment of inflammatory diseases, and of those acute and chronic complaints which are designated diseases of excitement." We have heard it objected, that a reviewer cannot fairly argue against a remedy he has not tried; and we acknowledge that we have not seen this drug given in the *form of powder* sufficiently often to judge of its effects. But, against this imagined necessity of pitching experience against experience, and case against case, we must protest, and beg to remind those gentlemen who use this shallow argument, that when any one is happy enough to discover a new remedy, or new virtues in one already known, that the *onus probandi* lies entirely with such discoverer, and it becomes the duty of the reviewer to examine carefully the evidence by which the pretensions to novelty are supported, and to point out wherein it is deficient.

It is true that we possess certain remedies which do command, to a great extent, the actions of the sanguiferous system, —such are digitalis, antimony, perhaps elaterium, and prussic acid: it seems, too, that when Mr. Alcock publishes his promised work on Mucous Membranes, we are to be made acquainted that the same power is likewise possessed by ipecacuanha. It appears, however, that the power of a remedy in controling inflammation, is not in the ratio of its command over the heart and arteries; for no other medicine possesses so marked an influence on these organs as digitalis, yet its power of lessening inflammatory action is by no means equal to that of antimony, although antimony cannot be made to govern the pulse with nearly so much certainty as foxglove: and, consequently, we regard Mr. Haden's reasoning as inaccurate, when he argues that colchicum, possessing great power in controling the action of the heart and arteries, is "therefore" capable of subduing inflammatory action. None of these remedies, we suspect, however, is capable, at least in this country, of being substituted with safety for blood-letting; and a medicine capable of really fulfilling this indication, is still a great desideratum in physic. We must confess, however, that we scarcely believe any such medicine exists; and still less do we expect to see any individual drug controling the various forms of inflammatory action, modified as it is in all its phenomena by the structure it attacks and by other adventitious circumstances.

The author, however, seems to think differently; and his own opinions, as well as those of his father, will best appear from the following quotations:

"I intend, in this essay, to offer to the profession the colchicum autumnale as a most powerful means of subduing increased or irregular action, or what we call inflammation in the constitution; and to show its powers of lessening the necessity for employing more hurtful remedies, such as bleeding, in acute cases of disease."

"If it be given every four hours, until it produce an abundant purgative effect, the pulse will become nearly natural, from being either quick and hard, or slow and full. This frequently happens even before purging has taken place; and the effect is so certain, that I never bleed, unless inflammation exists to an alarming degree in a vital part, and then never more than once.

"Fevers and inflammations so removed never require the use of tonic medicines during convalescence: the patients, indeed, generally appear to be as well as though they had not been at all the subject of disease; and, although it sometimes happens that a recurrence of symptoms take place, it is in a much milder degree, and the new disorder is always immediately removed in a few hours, by a very little of the same treatment."

In order to illustrate these doctrines, several cases are related, both of acute and chronic diseases, treated with this medicine; some of which are good, but scarcely any unexceptionable, as there is very seldom such a detail of symptoms as to enable the reader to judge for himself of the complaint; and, in most instances, the drug was combined with other remedies of acknowledged power. Besides, there are many of these cases so indefinite that, as they lead to no conclusion for or against the argument, we are at a loss to discover the object of their insertion. At pages 42 and 43, the three following cases appear in succession.

"Mrs. E. aged 30, was suffering from a common catarrh. She took six five-grain doses, and a small quantity of common opening medicine, in the morning, for a few days: after which, she took a bitter opening draught daily, on account of an old stomach affection; but was otherwise well.

"*October* 11*th.*—Mrs. L. aged 55, a corpulent person, who had suffered for some time from a slight degree of general feverishness, attended by heat at the stomach, and an itching eruption on the skin similar to urticaria. A *slight mercurial*, with *ipecacuanha,* was ordered for bed-time; and five grains of colchicum, with a drachm of sulphat of potash, in warm water, for the morning. A restricted diet was also recommended.

"26*th.*—She has continued to take the medicine, and is now nearly well.

"*October* 12*th.*—Mr. P. An old case of deranged digestive organs, with a white furred tongue, some heat of the skin, and considerable itching when warm in bed. Seven grains, with sal polychrest, were ordered to be taken every morning.

"26*th.*—He was better, but far from recovered: he had, however, not taken his medicine very regularly."

"Mr. B.'s servant, aged 16. This was a similar case to the last. The patient was going into the country, and eight powders were given to him, with directions to take one three times in the day. *No report* has been received of his progress.

"Mr. G.'s servant. Four powders were here given, for *a whitlow;* but, as the abscess was freely opened, the writer does not lay much stress on them as being the efficient means of relief."

"Mr. P. a baker, aged 30, applied for a *swelled face*, produced by the irritation of a portion of decayed tooth acting on the gum, in consequence of the habit being predisposed to fever. The piece was removed; but his tongue was much furred, and in the evening he had a severe attack of fever. Calomel and purging medicine were given; but, as he was very little better on the succeeding day, the colchicum was ordered. On the day but one after, he was met wheeling his barrow along the street; and had discontinued his medicine after the fourth dose. He was quite well on the sixth day."

Now, we can have no objection to the author prescribing colchicum in whitlow and tooth-ach; and, provided opening the abscess and extracting the carious tooth be premised, as in the examples just quoted, we have little doubt of the treatment being found efficacious.

Several cases are given of what is called "common fever," treated with colchicum; but the symptoms are not detailed. Mr. Haden, however, does not trust entirely to colchicum in idiopathic fever, but gives an instance, which we shall quote, of this disease *cured* by that remedy.

"For a reason which is mentioned afterwards, I have not hitherto trusted to the single use of colchicum in what we call idiopathic fevers; but, in the following case, it cured the complaint, and acted the part of an opiate, after calomel and common purging medicine only gradually and imperfectly relieved the symptoms. The colchicum, however, was pushed rather too far.

"*Case of simple fever treated by colchicum.*—Mr. Coates, aged 35, and weak, applied, with symptoms of common fever, on Sept. 28, 1819. Had had it for some days. The excitement was not fully developed, and the chief symptom was head-ache, which was rather severe. Ordered calomel and antimony, followed by black dose. Tongue was covered with a dull, dirty, brownish-grey, but smooth, slimy coat.

"29.—Was somewhat easier; had been purged. Rep. pil. et haust.

"30.—Head not much better; tongue beginning to clean at the edges; had been purged. Colchicum et sal polychrest. ter die.

"*October* 1.—Much better; was tranquillized in a very remarkable manner. Tongue much cleaner at the edges; great sweating produced; had been purged. Continue.

"*2d.*—Still better. Continue.

"*3d.*—Not quite so well, the medicine beginning to disagree: it had created sickness and general uncomfortable feelings. Sweating and purging continued. Stool dark, and in pieces; tongue with a small quantity of the dirty appearance in the centre, but the clean edges becoming covered with a thin white scurf, certainly from irritation on account of the over-dose of colchicum.

"This patient, from this time, required small doses of opening medicine for some days; when he was able to resume his common occupations."

Now, in perusing this case, the reader cannot but be struck with the very unsatisfactory detail of symptoms; for, though it is stated to have been fever, there is no mention, from first to last, of the state of the pulse; and of the skin we are only informed, that sweating followed the exhibition of the colchicum. With regard to the treatment, the medicine said to have cured the disease was not begun till, by repeated doses of calomel, with antimony and purging draughts, the tongue had begun to clean; a state of matters certainly very promising for any favourite remedy to play its part; and, had bread-pills been exhibited, the patient's recovery would probably have gone on without interruption. But, on the contrary, colchicum was ordered, which in three days had produced such general derangement as to require its discontinuance; after which, the patient got well. Similar to this are some cases published by Mr. Rice, in the London Medical Repository for January, as a kind of supplement to Mr. Haden's pamphlet: in all of these, except one, calomel and other powerful medicines were exhibited; and in some bleeding, both general and local, had recourse to. Nothing can show more strongly how unfit medical men, who have taken any particular remedy under their especial protection, are to judge of its real effects. We beg not to be misunderstood: we have ourselves employed, and seen others employ, colchicum largely, and, in certain complaints, with unequivocal advantage; and it is only when the author seriously proposes it as a substitute for bleeding, and other powerful means, in acute inflammation, that we must refuse our concurrence in his opinions, because they are not supported by sufficient evidence. We fear, too, lest the reputation of this excellent medicine should suffer from having virtues ascribed to it which will not, we believe, stand the test of experience.

There is another question of some importance in the therapeutic history of this drug: we mean, whether its purgative operation be or be not connected with its curative effect. Mr. Haden argues in the affirmative; and on this head, also, we differ from him. We have been in the habit of prescribing colchicum during the last three years, and have lately witnessed its exhibition on an extensive scale at one of the public institutions in this town. The result of our observations has been, that, when uncombined with laxatives, it does not induce purging nearly so often as seems to be generally supposed; and, as we never could perceive that the occurrence of this circumstance was attended with greater relief than when no such effect was produced, we are now in the habit of immediately checking it. We must be understood as speaking only of the vinous tincture, not of the powder; but we presume that the fact of purging being connected or unconnected with the peculiar effects of the remedy, must apply equally to either form. In general, we are in the habit of beginning with twenty drops three times a-day, and increasing the dose five drops each day till the patient takes two hundred or upwards in twenty-four hours; and, while we daily witness good effects under this plan in gout, rheumatism, and affections of the mucous membrane of the air-passages, we seldom see any considerable purging induced by it. When, however, this does occur, we have found a few grains of magnesia, night and morning, of great use in commanding it; and the same advantage, we are inclined from some recent trials to believe, may be obtained from small doses of ipecacuanha.

While speaking of the action of this remedy, we may here remark, that the two writers who have volunteered as its advocates differ somewhat in their opinions; for, while Mr. Haden regards it as particularly useful in cases of *excitement*, Dr. Williams of Ipswich, on the other hand, regards it as beneficial chiefly in complaints of the *asthenic kind*.

It is but justice to Mr. Haden to say, that he does not seem quite satisfied with his own work. "It appears," he says, "that this is a very imperfect account of the medicinal effects of the colchicum autumnale. It fails in discriminating accurately the forms of acute disease in which the remedy acts most efficaciously: it does not point out those in which it is useless; it refers almost entirely to the use of the remedy when given only in one state and in one combination; and, therefore, the advantages which may doubtless be derived in many forms of complaint by giving it in conjunction with other kinds of medicine, are scarcely hinted at." Were authors in general to point out the errors of their works with as much fidelity as is done here, it would often save the reviewer an ungracious task.

*General Elements of Pathology.* By WHITLOCK NICHOLL, M.D. M.R.I.A. F.L.S.; of the Royal College of Physicians, London; Member of the Medical and Chirurgical Society; Corresponding Member of the Association of King's and Queen's College of Physicians, Dublin. 8vo. pp. 233. Callow, London, 1820.

Εμοι δοκέει ἀρχὴ μεν ὀυν ὀυδεμία ἐιναι τȣ σώματος, ἀλλα πάντα ὁμοίως ἀρχὴ, καὶ πάντα τελευτή· κύκλȣ γαρ γραφέντος, ἀρχὴ οὐχ εὑρεθη· καὶ των νοσηματων ἐστι παντὸς ὁμοίως τȣ σώματος.—HIPPOCRATES *de Locis*, &c. cap. i.

THIS work has neither "Preface," "Introduction," nor "Preliminary Observations;" but, in compensation for the loss of these fashionable preludes, the author adduces, as an epigraph, an extract from the writings of BAGLIVI, in which he exposes his intentions, and designates pretty well, we think, the merits and utility of the book.

"*Hoc opusculum ut in publicum ederem, non fecit profecto inanis, ac popularis auræ captandæ cupiditas, sed eo adductus sum, ut multis meorum æqualium hinc inde errantibus viam monstrarem, et aliquantulum munirem.*"

We like so well the abrupt manner in which the work commences, and we have been of late so heartily nauseated by the *jejune* and trite *exordia* which most of our modern critics, especially those of a neighbouring continental nation, append to their "Reviews," and lavish in a way that might alone lead a man of ordinary shrewdness to anticipate their inanity, and suspect that they must be constructed at no great degree of expence, that we are glad to have so good an apology as the example given us by our present author, for omitting a preamble about the utility of systematic works on science;—the relative value of those constructed solely of precise inferences from acknowledged facts, and those framed by adopting, in the first instance, partly on analogy, some general principles under which the requisite facts may be arranged with tolerable grace and facility;—some congratulations with our contemporaries on the establishment of the inductive mode of philosophizing now in vogue, and on the discredit of speculative reasoning; with a few complacent hints that we are the finest specimens of human nature, and that our forefathers, especially the "ancients," knew not how to use their intellectual faculties;—we are glad that we have so good an apology for omitting all this, and for entering at once on an exposition of the work before us.

The book commences with an "*outline of the human economy*," in which the author gives only a simple demonstration of sensible phenomena, in the order in which they occur, as the functions are generally performed in the healthy state of the system, without entering on any considerations respecting their causes, or the means by which they are effected, beyond such data as are evident to the senses, or obviously exist in the mechanism of the organs by which those phenomena are developed. He begins by saying, that "Living man consists of an organized body, to which are attached life and intellect. The organized body may be described, as consisting of a system of supply and waste; of a nervous system; of various assemblages of contractile fibres, which are called muscles; and of a fundamental structure, which consists of bones, of cartilages, and of the varieties of membrane. The system of supply and waste, consists of the vascular system, and of its two appendages,—namely, the alimentary canal, and the pulmonary air-cavities, (air-*cells.*) The vascular system consists, of the heart; of the arteries; of exhalants; of secreting vessels, with their several ducts, reservoirs, and outlets; of sinuses and veins; and of absorbents."

Having described the distribution of the vascular system, the author then treats of the course of the blood, (which fluid is not, we cannot discern wherefore, mentioned amongst the constituents of "living man,") and the changes produced in it in the lungs, and by means of the fulfilment of the functions of the systems of *supply* and *waste*. The phenomena of digestion, and the functions of the absorbents, are next described; when the author remarks that, "although the office of all absorbing vessels is so far the same that they all take up, or receive, various kinds of matter throughout the body generally, yet may they be considered as performing three sets of offices. In the first place, they take possession of fresh supply, which is for the first time entering the system; as when fresh matter is absorbed from the internal surface of the alimentary canal, or from the pulmonary air-passages, or from other passages having external outlets, or from the surface of the body. In the second place, they take possession of fluids which form a part of the waste; as when exhaled, or secreted, fluids pass into absorbents. In the third place, absorbents take possession of matter which has previously been deposited from the vascular system, and which has formed a portion of the structure of the body; as when the more solid parts of the body are removed by absorbing vessels."

As the author, in this part of the work, treats of the phenomena of the body in the state of health alone, it is obvious that he asserts that the solid structures of the body are in that state undergoing mutation: a proposition which is not at all satisfactorily established, as the observations and reasonings of WINTRINGHAM, LISTER, BOHN, SCHELLAMER, GIBSON, and FRING, sufficiently indicate.

As this work is constituted, almost exclusively, of a series of aphoristic sentences that can be of utility only by furnishing the means by which a reflective man may attain to some general views of the functions of the human economy, it might, with reason, have been expected that the materials, which are in fact but the "*Elements*" of a system of pathology, had been only such as are of undisputed validity, or that the author had more strictly distinguished the positive facts he adduces, from inferences which are merely probabilities. There are, however, but few specimens of the fault here designated, though this section of the work contains another, where it is said that "some of the extreme branches of this artery (the aorta) terminate in exhalants." It has never been demonstrated that the arteries any where terminate in exhalants: on the contrary, it is rendered highly probable, by the experiments and observations of Leuwenhoek, Cowper, Malpighi, Spallanzani, Ruysch, De Graaf, Vieussens, and many other able anatomists, that the extremities of the arteries are every where continuous with the veins, and that the exhalants are, exclusively, given off from the sides of the arteries throughout the greater part or the whole of their course: a view of the subject that is not at all opposed by the results of the experiments of Cruickshanks, Haller, Nuck, and several other anatomists, who have filled the lymphatics of a part by an injection of the arteries of it.

This section goes on to treat, in a very general way, (the author referring to a work which he published a short time since, entitled "A Sketch of the Economy of Man," for details respecting the physiology of the body,) of the phenomena of respiration, the action of the heart, the properties of the arteries, and the influence of the nervous system; and this short section concludes with the first of a series of remarks that are illustrative of the motto to the work.

"Thus it appears that the several parts of the body are intimately connected with, and dependent upon, each other. For, the functions of the vascular system would cease, if those of the nervous and muscular systems were suspended; the functions of the nervous system would cease, if the offices of the vascular system were suspended; and the muscular system would be inert, were it not for the influence which it derives from the vascular and from the nervous systems."

Having thus treated of the phenomena of the human economy in the state of health, the author proceeds to consider, in a similar, though more particular, manner, those which occur in the deviations from that state; and he discusses them in an order, in regard to their origin, correspondent with that which he has adopted in passing in review the subjects of the former section. The first relates to the "*quantity of the blood.*"

It being part of our duty to give a specimen of the author's manner of treating the subjects of his disquisitions, we shall transcribe the whole of the present section : it is one of the most concise ones in the book, and it will enable the reader to form an adequate idea of the general character of the work ; for each of the subjects of it, to be presently enumerated, is treated on in a precisely similar manner.

" If there be an increase of the mass of blood, unaccompanied by an increase of the action of the heart, either as to force or as to frequency, the blood, although its quantity is increased, will have its momentum diminished.

" In such a case, the tonicity of arteries being less strongly opposed, the capacity of those vessels,—of the smaller arteries especially, (in which, as I have already stated, the degree of tonicity is greater, in proportion to the size of the vessel, than it is in the larger arteries,)—will be lessened, so that an increased quantity of blood, and an increased proportion of the general mass of that fluid, will be contained in the larger arterial trunks, in the veins, and in the cavities of the heart. The whole round of the circulation will be obstructed. A diminished quantity of blood will pass into secreting vessels; but exhalation may be increased, owing to the lessened momentum of the blood. The action of the heart will consist of slow, feeble, contractions, or of ineffectual flutterings. The pulmonic process will be imperfectly performed, and respiration will be laborious and hurried. The quality of the blood will be rendered unnatural.

" The circulation of blood of an unnatural quality through the cerebral blood-vessels, will induce disorder of the cerebral structure, and of its functions. From the obstructed state of the whole round of the circulation, congestion of blood may take place in the cerebral veins and sinuses, whence may ensue inordinate compression of the cerebral substance, and, consequently, disorder of the whole of the nervous system. Or, should the cerebral exhalants pour out an increased quantity of fluid, in consequence of the obstructed state of the cerebral veins, inordinate compression of the brain may also be produced, unless the exhaled fluid be removed in an equally increased proportion. The sensibility of nerves will be lessened, in consequence of the smaller quantity of blood which circulates through the blood-vessels in their vicinity, as well as from the altered state of that blood, and also in consequence of the disordered state of the cerebral structure with which their cerebral extremities are connected. The production of nervous power will be lessened, and the expenditure of it will be disturbed. The generation of heat, and the capacity of the body for heat, may be lessened. Muscular action will be feebly, or imperfectly, or irregularly, performed. Sensation will be sparingly, or imperfectly, produced. The depressing passions will prevail. The faculties will be feebly, or imperfectly, exerted. All these altered conditions of the several parts and functions of the economy, will produce disorder of the alimentary canal; consequently, digestion and chylification will be imperfectly performed, and the supply will be diminished and depraved. The performance of the pulmonic process will become still more deranged, in consequence of the disordered state of the nervous system and of the intellect, owing to the direct effects which such disordered states produce on the respiratory muscles. In short, cause and effect will act reciprocally throughout the whole economy, producing complete disorder of every part, and of every function.

"If the mass of blood be increased, and if the action of the heart be also increased, so as to propel the greater mass with freedom, the momentum of the blood will also be increased.

"In such a case, the tonicity of the smaller arteries being more powerfully opposed, these vessels will yield more readily to the current of the blood; they will therefore receive a greater quantity of that fluid, and, consequently, an increased quantity must pass by their terminations. The quantity of secreted, and of exhaled, fluids will be increased. The blood will flow with greater force, and in greater quantity, throughout the whole round of the circulation. As an increased quantity of the blood will pass, in a given time, through the pulmonic circuit, respiration must be more quickly performed; otherwise, that fluid will not duly undergo the pulmonic process. The sensibility of the nervous system may be increased, and the functions of that system may be more freely performed. Nervous power may be more freely produced. Heat may be generated in increased quantity. Muscular action may be performed with increased energy, and to a greater extent. The depressing passions will subside, and the livelier passions will predominate. These altered conditions of the several parts and functions of the economy, will affect the state of the alimentary canal, will alter the processes of digestion and of chylification, and will influence the action of that canal.

"It may happen that the increased quantity of blood received by the cerebral blood-vessels, may induce what I have termed *erethism** of the cerebral substance; or it may induce inflammation of that substance. Or it may happen, either from simple plethora of the cerebral blood-vessels, or from erethism of the cerebral structures, (whether the state termed inflammation be or be not present,) that an increased flow of fluid from the cerebral exhalants may take place, producing inordinate compression of the cerebral substance. Or simple plethora of the cerebral blood-vessels may cause the brain to be inordinately compressed. Such a state of the cerebral structures, in what way soever induced, will cause disorder of the whole nervous system.

"If the quantity of blood in the vascular system generally be lessened, the heart (having a smaller mass to propel, and probably receiving a slighter stimulus than before, and probably, also, having the tone of its fibres diminished, owing to the lessened quantity of blood that flows through the coronary vessels,) will act with less force or with less frequency, or its actions may consist of feeble contractions quickly repeated. The blood, then, will either flow in a languid manner, or it will be rapidly hurried through the round of the circu-

* See *Remarks on Affections of the Cranial Brain in Infants.* Transactions of the Association of the King's and Queen's College of Physicians in Ireland, vol. iii.

lation. The momentum of the blood will be lessened; consequently, the smaller arteries will more successfully oppose the entrance of that fluid into them. This increased resistance of the smaller arteries may still keep up, in the larger arteries, a quantity of blood nearly equal to that formerly contained in them. The quantity of secreted fluids will be diminished; but, if the blood flow in a feeble manner, the usual quantity, or even an increased quantity, of fluid may pass from the open mouths of exhalants.

"If the blood, although its quantity be much lessened, flow with increased rapidity, the velocity with which it is distributed may, in some respects, compensate for its diminished quantity: still, as the actual quantity of it is lessened, the quality of the fluids separated from it in secreting vessels will not be the same as before, even supposing that those fluids are secreted in the usual quantities.

"As a smaller quantity of blood will, in the case now under consideration, flow through the blood-vessels of the cranial and spinal brains, the functions of those structures may be, more or less, suspended or destroyed. The nervous power will be sparingly produced, and it may be irregularly distributed. The sensibility of the nervous system, generally, will be lessened. The temperature of the surface of the body will be lessened. Sensation may be feebly and scantily produced. The livelier passions may be wanting, and the faculties may be feebly and imperfectly exerted.

"It may happen, in consequence of the languid motion of the blood, that an increased flow of fluid may take place from the cerebral exhalants, in which case inordinate compression of the cerebral substance may arise.

"All these altered states of the nervous system, of the muscular system, and of the intellect, will re-act upon the system of supply and waste, and upon the parts appended to it, increasing the disorder of these several parts.

"The quantity of the general mass of the blood is dependent upon the quantity of supply which that fluid receives from absorbing vessels, and upon the extent of the waste which it suffers from the processes of secretion and of exhalation."

Amongst the objects (besides that already mentioned) which we have had in view in making the foregoing transcription, one is that of acquainting the reader that an abstract analysis of this work is not to be expected in this article,—it must be evident that it will not admit of such a production; and another is that of showing the grounds on which we establish the principal critical remarks we have to make on these "Elements" as a medium of instruction in pathology. We think they are qualified to fulfil, to a very considerable extent, the intentions expressed by the author in the epigraph which we have quoted. It is especially to those who approach somewhat towards "*æquales*" of the author that the work will be of much utility; and it will prove useful to them principally, by furnishing precise, and, with but a few probable exceptions, correct, ideas of the phenomena of the human economy, in its various states of disease, derived, generally, from views equally minute, perspicuous, and comprehensive, with those manifested in the passage above transcribed. We do not mean to state that it is not qualified to benefit the young student: on the contrary, we earnestly recommend a perusal of it immediately after he has attained a moderate degree of anatomical information; but a student might retain the whole of the book in his memory, and yet, if he were not otherwise instructed, be at a loss to know the nature of almost any one of the diseases he would meet with in walking round the wards of an hospital, in consequence of its not presenting any thing like pictures of the distinct groups of symptoms which ordinarily accompany individual forms of disease. There is, in the work, a section devoted to "general Inferences;" but these are not calculated to supply the deficiency here alluded to. As nothing respecting symptomatology is advanced, no notice, of course, is taken of the peculiarities in the phenomena and effects of disease dependent on peculiarities of the structure—as the mucous, fibrous, and serous, membranes,—in which it is seated. We are aware that the author has not professed an intention of adapting his work for the instruction of students, properly; and our object in the foregoing remarks has only been to express somewhat fully our opinions respecting the extent of the utility of these "Elements." Pursuing our intention, then, we may further observe, that this work is qualified to be very useful to the young student, by supplying him with precise ideas of the phenomena of the human economy, devoid of any general abstract notions of disease, which can only prove embarrassing in the first instance; but, when he has acquired such ideas, he should leave the study of it for clinical observation and treatises comprising histories of individual diseases, and more abstract and general notions in pathology, and revert to it when these have been duly attended to, as a source of the most important of the essential facts concerned in the origin and progress of diseases, that is characterized by the qualities which we have already conceded to it. The study of it will also tend to generate habits of more minute as well as more extensive circumspection, in his clinical observation, than he might, probably, fall into without such a guide. Although it possesses such qualities, we, however, suspect that the book will never become a popular one, (and that it should be such, does not appear to have been the intentions of the author:) a great proportion of the medical men, to whom such a treatise may prove useful, like books that require less mental exertion on their part, in order that inferences may be obtained from them applicable to the purposes of practice. There will not, however, be wanting those who will think that Dr. Nicholl has rendered an important service to medical science, by forming a summary of the "Elements of Pathology" acknowledged at the present day, that, although it wants the lustre and highly important uses of general principles founded on abstract theory, has the advantage of being almost wholly devoid of hypotheses. We say *almost* wholly devoid of hypotheses, because, besides the notions already alluded to respecting the functions of the absorbents, it comprises some opinions on the nature of inflammation that have the character just indicated. These will be noticed hereafter.

The work being, as it appears to us, what the author intended it to be, it would be impertinent in us to advert in a particular manner to the superior value of treatises which comprise more general or abstract notions of the relations of morbid phenomena; but, there is one very important related series of such phenomena, the laws regulating which are really "*Elements*" of Pathology, that is not at all indicated in the work of Dr. Nicholl, and which, indeed, could not, perhaps, have been considered without a deviation from the plan he has followed in his dissertations. We allude to the phenomena termed *sympathetic*. A great deal of vague and silly declamation has been uttered against the use of the word sympathy in pathology, by persons ignorant of its etymology, and of the sense in which it has been employed by medical writers. No intelligent practitioner doubts of the occurrence of related morbid phenomena, the connection of which is not explicable by any principles founded on the anatomy of the body, or by a generic mode of function, and which occur too regularly to be attributed to chance; and the term sympathy is applied to the relation in question, not with the view of *explaining** it, but merely to express that relation; as the word *attraction* was employed by NEWTON to designate a *result*, without pretending to state whether its cause existed in the subjects of it, or in some other bodies extraneous to them. We shall notice this subject more particularly in an ensuing part of this Journal. At present we direct our attention to the part of the work before us where our last transcript terminates, for the purpose of passing in review the rest of it, though we know not what more we can do than designate the particular subjects of the several sections of which it is constituted, and

* This word is here used in its ordinary sense, though, in fact, *explanation* is but the assimilation of phenomena of which we know but little, to others with which we think we are better acquainted: thus, after having ascertained that the centripetal force of the moon and *weight* were conformable, in their effects, with the same laws, Newton affirmed their common cause to be *gravitation*. We fancy we have some ideas of gravitation; and so, by this *explanation*, we think we are enabled to understand in some degree the cause of the centripetal force of the moon.

point out the opinions contained in them that are, more or less precisely, peculiar to the author: and we wish to have it understood, that when we cite the title of a section without making any remarks on it, that section contains no such opinions as those just designated; the characteristics of them being the same with those of that on the "quantity of the blood."

The next section treats of the "*action of the heart*:" then ensue others on "*secretion*," "*exhalation*," the action of the "*veins*," and of the "*absorbents*." From that on the latter subject we extract the following remarks, as the doubts they involve of the direct influence of certain medicines on the action of the absorbents, may be regarded as notions proper to the author. The best alternatives, in the way of inferences, that we are acquainted with, on this point, are stated by Mr. Pring, in his essay on the absorbent system, (pp. 33—45.)

"It is easy to comprehend," says Dr. Nicholl, "how medicines, which are admitted into the alimentary canal, may get into the mass of circulating blood; and how they may, consequently, affect the condition of that fluid, and the state of the vessels in which that fluid is contained. But we cannot well conceive how such medicines can act upon absorbents, unless we suppose that they are poured forth from exhalants, and that they then act upon the mouths of the absorbing vessels, or that they act upon absorbents through the medium of the nervous system. Digitalis and squill, when admitted into the alimentary canal, cause an increased secretion of urine, as do other medicines belonging to the class diuretics, which also produce the removal of dropsical accumulations. But neither digitalis nor squill, nor other diuretic medicines, procure the decrease of dropsical accumulations, unless they cause an increased production of urinary fluid, or produce an evacuation of watery fluid by some other outlet. It seems probable, therefore, when the exhibition of digitalis, of squill, or of any other diuretic medicine, is followed by a decrease of any dropsical accumulation, that such remedy must produce this effect by causing a suspension, or a diminution, of the process of exhalation, either by its direct effect upon exhalants, or, as is more probable, by causing an increased separation of fluid by the kidneys; in consequence of which, exhalation is diminished or suspended. For it is plain that, if exhalation be diminished or suspended, while the absorption of the effused fluid continues to the usual extent, a diminution of the quantity of accumulated fluid must ensue. In short, we seem to be warranted in considering that, when diuretic medicines procure the removal of dropsy, they produce this effect by their direct influence on the process of secretion, and by their consequent influence on exhalation, rather than by their influence on absorbents."

The author does not altogether deny that stimulants may directly influence the absorbents, and thus increase absorption; for he says (in his aphorisms respecting absorption) that it seems probable that those vessels possess a contractile structure, and that the application of stimulants may cause increased contraction of that structure.

The ensuing sections relate to the "*alimentary canal*," the "*pulmonic process*," the "*cranial brain*." In the last-mentioned section, the author, after BORDEU and some other writers, employs the term *erethism* in the same sense as *excitement* has ordinarily been used by the generality of pathologists. Erethism of the brain, he considers, may exist without any alteration of the state of the vascular system of the part; but it is disposed to cause an increased quantity of blood to flow through those arteries, and this combination of phenomena, he believes, constitutes inflammation. This is a point of doctrine that wants confirmation: it is by no means proved that an increased quantity of blood flows (in a given time) through the arteries of a part in a state of inflammation; and there are not wanting forcible arguments for a converse inference; for some of which we may refer to our review of Dr. Hastings' Treatise on Bronchitis.

The author then treats on the "*spinal brain*," and the "*sensibility of nerves*."

"There are," he says, "two kinds of habits, or temperaments, in each of which there is an unusual degree of sensibility of the nervous system generally. In the one, there is a great disposition to the production of heat; in the other, there is a great disposition to a diminution of temperature. In the one, any sudden alarm, or agitation of mind, or bodily pain, induces great increase of temperature, which is attended with dryness of the skin and with quickened action of the heart: in the other, such mental affections are attended with great coldness of the body, diminished action of the heart, and profuse perspiration. In the one, the skin is scarcely ever moist, and the pulse is rarely slow: in the other, the skin is generally damp, and the pulse is rarely frequent. In the one, there is great muscular strength and activity; while the other is delicate and feeble. In the one, there is generally an active, acute mind, whose energies are called forth by passions or by pain: in the other, there is rather a feeble, dispirited state of mind, which by powerful passions, or by pain, is rendered inert."

The "*nervous power*," "*temperature*," and "*muscular action*," are next discussed, (the last subject in a very extensive manner;) and then the dissertations of this class conclude with one on "*intellect*:" after which follows a section devoted to "*general inferences*." The author here, in the first place, adduces a series of remarks, the chief object of which is to show the mutual influence of the various phenomena of the economy on each other,—or, in other words, to demonstrate the truth of his *motto: It appears to me*, (says HIPPOCRATES,) *that no one of the phenomena of the human body can be regarded as the prin-*

*ciple, or source of origin, of the rest; but that all are equally principles and all final results. We cannot mark out, in a linear circle, any point at which it begins.* Dr. Nicholl then adduces some reflections, of a general character, on the nature of disease; the tendency of which is to show the impossibility of constructing any nosological system that is not qualified, to a certain extent, to lead to error, by confining our views to merely partial assemblages of the phenomena of morbid states, and, using the metaphor of Galen, by leading us to mistake shadows for the objects to which our attention should be directed. He then, alluding especially, it would appear, to the arrangements of Sennert and his followers, and Pinel, observes that—

"There are two ways in which the bodily structure of man may be described. We may speak of it as being composed of distinct sets of structures, as of those which are comprised under the terms vascular system (and its appendages), nervous system, muscular system, and basal structure; which latter term comprehends the bones, the ligaments, and the varieties of membrane: or we may treat of it, after the manner of geographers, as consisting of the head, of the neck, of the thorax, of the abdomen, and of the upper and lower extremities; subdividing each of these parts, and enumerating the several contents of each. An equal degree of mischief may result from each of these modes of considering our structure; for, whichever mode we adopt, we acquire a habit of considering each portion which we enumerate, as a distinct and insulated part. The consequence is, that when any deviation from the healthy state occurs in any one of the sub-divisions which we have made, our attention is fixed upon the diseased condition of this particular portion of the body, while every other portion is supposed to preserve its former healthy state.

"Thus, those who have adopted the first mode of considering our bodily structure, speak of diseases of the nervous system, diseases of the vascular system, and diseases of the muscular system. In so doing, they are incorrect; in as much as a disordered state of either of these systems implies also a diseased condition of each of the other systems.

"Those, on the other hand, who adopt the latter mode of regarding our bodily fabric, speak of diseases of the head, of the chest, of the abdomen, of the skin, &c. They subdivide these into diseases of the cranium, of the cranial brain, of the lungs, of the heart, of the liver, of the stomach, of the bowels, of the spleen, of the pancreas, and so on. These persons act incorrectly, also, because, in as much as, in each part which they enumerate, there are portions of the general nervous system and of the general vascular system, it is evident that each part cannot be considered as a distinct, insulated republic, but as a constituent portion of the general commonwealth, whose health is dependent upon a certain condition of every portion of the body.

"As the several animated beings of our globe have been reduced into classes, orders, genera, and species, in which they have had their several places assigned to them, from some leading characteristic in their form, their habitation, or their economy, so has an attempt been made to form a similar arrangement of diseased states.

"If we examine the different arrangements which have been framed by nosologists, we shall find that each class, and indeed each sub-division of each class, is, for the most part, founded upon some leading symptom, or upon the preponderance of the deviation from the healthy state occurring in one particular part, or in one particular function. Since, then, such symptom, or such preponderating deviation, may, as we have seen, be the result of an altered condition of any part or function of the economy, it follows that no one of such classes, nor any one of the sub-divisions of either class, can comprehend the result of any peculiar primary deviation from the healthy state; for the same primary deviation from the healthy state may induce conditions of the economy which are comprehended under each of the classes, and probably under each of the sub-divisions of each class. In other words, diseased states which are arranged under a variety of distinct heads, may alike be the consequence of the same primary deviation from the healthy state: and, on the other hand, each diseased state contained in each class may alike arise from an altered condition of any one part, or of any one function, in the economy."

The nosology of Cullen is then more particularly examined, in pursuance of the foregoing views; and, finally, the ills that arise in practice from a devotion to such classifications, are pourtrayed with equal force and precision.

Three Appendices are attached to the work: the first of which consists of an analysis of the phenomena of fever, constituted after the manner of the sections in the body of the work, and, consequently, is not adapted for an abstract exposition; nor is it a fit subject for any other judicial remarks than such as we have made on the author's dissertations in general. We may refer to the *Proemium* to the present volume of this Journal, for details on this subject. The second Appendix comprises a disquisition on inflammation, of a similar character to the foregoing one; only that this comprises the hypothetical opinion respecting the state of the circulation in an inflamed part, noticed on a former occasion. The last is constituted of a series of "Aphorisms respecting Absorption." Of the merits of this part in particular, we could only express the same opinions with those we have adduced respecting the work in general.

